# Chiari Malformation Type I: A Case-Control Association Study of 58 Developmental Genes

**DOI:** 10.1371/journal.pone.0057241

**Published:** 2013-02-21

**Authors:** Aintzane Urbizu, Claudio Toma, Maria A. Poca, Juan Sahuquillo, Ester Cuenca-León, Bru Cormand, Alfons Macaya

**Affiliations:** 1 Pediatric Neurology Research Group, Vall d'Hebron Research Institute, Universitat Autònoma de Barcelona, Barcelona, Spain; 2 Department of Neurosurgery, Research Unit Vall d'Hebron University Hospital, Universitat Autònoma de Barcelona, Barcelona, Spain; 3 Neurosurgery and Neurotraumatology, Research Unit Vall d'Hebron University Hospital, Universitat Autònoma de Barcelona, Barcelona, Spain; 4 Departament de Genètica, Facultat de Biologia, Universitat de Barcelona, Barcelona, Spain; 5 Biomedical Network Research Centre on Rare Diseases, Barcelona, Spain; 6 Institut de Biomedicina de la Universitat de Barcelona, Barcelona, Spain; Universität München, Germany

## Abstract

Chiari malformation type I (CMI) is a disorder characterized by hindbrain overcrowding into an underdeveloped posterior cranial fossa (PCF), often causing progressive neurological symptoms. The etiology of CMI remains unclear and is most likely multifactorial. A putative genetic contribution to CMI is suggested by familial aggregation and twin studies. Experimental models and human morphometric studies have suggested an underlying paraxial mesoderm insufficiency. We performed a case-control association study of 303 tag single nucleotide polymorphisms (SNP) across 58 candidate genes involved in early paraxial mesoderm development in a sample of 415 CMI patients and 524 sex-matched controls. A subgroup of patients diagnosed with classical, small-PCF CMI by means of MRI-based PCF morphometry (n = 186), underwent additional analysis. The genes selected are involved in signalling gradients occurring during segmental patterning of the occipital somites (FGF8, Wnt, and retinoic acid pathways and from bone morphogenetic proteins or BMP, Notch, Cdx and Hox pathways) or in placental angiogenesis, sclerotome development or CMI-associated syndromes. Single-marker analysis identified nominal associations with 18 SNPs in 14 genes (*CDX1*, *FLT1*, *RARG*, *NKD2*, *MSGN1*, *RBPJ1*, *FGFR1*, *RDH10*, *NOG*, *RARA*, *LFNG*, *KDR*, *ALDH1A2*, *BMPR1A*) considering the whole CMI sample. None of these overcame corrections for multiple comparisons, in contrast with four SNPs in *CDX1*, *FLT1* and *ALDH1A2* in the classical CMI group. Multiple marker analysis identified a risk haplotype for classical CMI in *ALDH1A2* and *CDX1*. Furthermore, we analyzed the possible contributions of the most significantly associated SNPs to different PCF morphometric traits. These findings suggest that common variants in genes involved in somitogenesis and fetal vascular development may confer susceptibility to CMI.

## Introduction

The Chiari malformations, first described in 1895 [1], are a group of disorders sharing the common feature of ectopia of the cerebellar tonsils, which are downwardly displaced through the foramen magnum. Chiari malformation type I (CMI) is currently defined by the observation, on cranial midsagittal magnetic resonance imaging (MRI), of a descent of the cerebellar tonsils across the foramen magnum of at least 3 mm [2]. In contrast with Chiari II and III malformations, in CMI the rest of hindbrain structures remain within the posterior fossa.

CMI causes neurological dysfunction by direct compression of the neural tissue at the craniovertebral junction or cerebrospinal fluid disturbances that give rise to syringomyelia or hydrocephalus. The most frequent symptoms are headache, ocular disturbances, vertigo, sleep apnea and lower cranial nerve signs, including tongue fasciculation, dysphagia or dysarthria. Motor and sensory symptoms derived from spinal cord disturbances associated with syringomyelia or scoliosis, [3], [4] as well as signs and symptoms mimicking pseudotumor cerebri are also often encountered. Although presentation typically occurs in middle age, it may start in childhood or infancy, where it is a cause of sudden infantile death syndrome [5], [6]. Conversely, cases of CMI can often be asymptomatic and tonsillar descent is often regarded as an incidental neuroradiological finding [7], [8]. The prevalence of CMI is estimated to be in the 1/1,000 to 1/5,000 range [9]. A retrospective analysis of 22,591 serial cranial MRI studies, using a conservative definition of cerebellar tonsillar herniation of ≥5 mm, showed the presence of CMI in 0.77% of the subjects and asymptomatic CMI in 0.11%, with an estimated prevalence of 1/1,280 [10].

**Figure 1:**
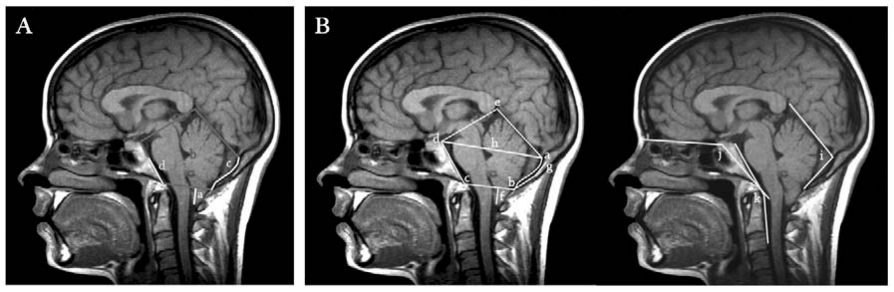
Typical neuroradiological findings in CMI. A) T1-weighted mid-sagittal MR image showing downward herniation of the cerebellar tonsils (a) and hypoplasia of the occipital components of the posterior cranial fossa (b): the supraocciput (c) and the basiocciput (d). B) Morphological measurements performed: length of tonsillar descent (f), supraocciput (g-b) and clivus (c–d) and foramen magnum diameter (b–c). The antero-posterior diameter of PCF (h) was inferred from a line running from the internal occipital protuberance (a) to top of the dorsum sellae (d). The PCF area was estimated as the polygon delimited by (a) (b) (c) (d) and (e) and the osseus PCF area as the one delimited by (a) (b) (c) and (d). Angular measurements: tentorium angle (i), basal angulation (j) and Wackenheim angle (k).

Regarding pathogenesis, different mechanisms are to be distinguished. In the classical form, a shallow posterior cranial fossa (PCF) is unable to house a normal hindbrain and hence the tonsillar herniation occurs (Figure 1A). Seminal experimental work by Marin-Padilla and Marin-Padilla [11] demonstrated that underdevelopment of the occipital bone and overcrowding of the cerebellum in a small posterior fossa are the fundamental defects in CMI [12], [13], [14]. This has been underscored by several radiological morphometric studies [3], [15], [16], [17], [18], [19]. However, cerebellar herniation may also occur in conditions such as cranial settling associated with connective tissue disorders, intraspinal hypotension following CSF leaks or lumboperitonial shunting, cord traction in tethered cord syndrome and intracranial hypertension caused by space-occupying lesions or hydrocephalus [20], where the PCF has a normal size and the term “secondary” CMI is preferred. In the classical form of CMI there is shortness of the basichondrocranium [3], [15], [16], [17], [19], [20], [21], [22], suggesting underdevelopment of occipital somites and pointing at insufficiency of the paraxial mesoderm as the central event in CMI pathogenesis [23].

Whether CMI mesodermal insufficiency is a primary (genetic) condition or occurs as the result of early gestational injuries, or even post-natal osseous growth disturbances [16] is unknown. However, a number of evidences suggest that, at least in a subset of CMI cases, genetic factors may play an important role, including CMI familial aggregation [9], [24], higher degree of concordance in monozygotic twins and CMI occurrence as part of Mendelian syndromes [25], [26], [27]. In the instance of Crouzon syndrome, a specific *FGFR2* mutation has been associated with CMI and syringomyelia in a small series of cases [28] and a *PAX2* mutation was found in two siblings with CMI and coloboma [29]. The only reported genome-wide linkage screen, performed in 23 CMI multiplex families, uncovered two loci on chromosomes 9 and 15, with maximal two-point LOD (logarithm of the odds) score of 3.3 on 15q [30]. To date, no single gene has been identified as causing CMI.

If heredity does indeed play a role in CMI, it is also possible that this may occur via a polygenic mechanism. Common genetic variants in genes involved in growth and shaping of the PCF should be among the first to be explored. A similar approach has been used in association studies performed in patients with Chiari malformation type II, where screening of genes critical for neurulation and neural tube closure disclosed some risk factors but no major causative gene (reviewed in [31]). In contrast, no previous association study has been carried out in CMI. We here sought to identify common variants associated with development of CMI among critical genes expressed during occipital somite, sclerotome and placental development.

## Subjects and Methods

### Ethics Statement

The present investigation was conducted according to the principles expressed in the Declaration of Helsinki and was approved by the Ethics Committee of the Vall d'Hebron University Hospital. All participants signed an informed consent prior to their participation in the study.

### Subjects

A total of 415 patients (42.2±5.9 years) with symptomatic CMI were recruited between 2004 and 2010. Three hundred and thirty seven patients were diagnosed and treated at the Division of Neurosurgery and the Pediatric Neurology Service of the Vall d'Hebron University Hospital in Barcelona. In all of them evaluation included direct anamnesis, physical exam, neurophysiological studies (somatosensory evoked potentials, brain auditory evoked potentials and polysomnography) and a cranial and spinal MRI study. For an additional 78 cases that were referred from two Spanish CMI patient associations, review of clinical summaries, radiological reports and telephone interviews were used to confirm the diagnosis, but direct review of the MRI studies was not always possible. Diagnosis of CMI was based on MRI demonstration of downward herniation ≥3 mm of the cerebellar tonsils on a midsagittal T1-weighted image in the presence of signs or symptoms indicating neural compression at the craniovertebral junction, syringohydromyelia, cerebellar dysfunction or intracranial hypertension

Since recent studies have clearly demonstrated that various mechanisms may lead to tonsillar herniation, we tried to identify the subset of patients with small PCF in an attempt to increase sample homogeneity. To this end we performed a morphometric PCF analysis in 211 cases with available digital MRI studies (Figure 1B). After applying a previously described logistic regression model based on several PCF measurements performed in CMI cases and a control population [32] we identified a subgroup of 186 patients representative of classical CMI, i.e., with underdeveloped PCF.

Clinical data was retrieved from an in-house patient database devoted to Chiari malformations and related disorders, a clinical research repository that included detailed information about the medical history, family history, symptoms and signs, treatment and any relevant associated conditions.

The control sample was composed of 524 healthy blood donors (42.6±14.4 years), sex-matched with the case sample and recruited at Vall d'Hebron University Hospital in Barcelona. To minimize ethnic heterogeneity only Caucasian subjects of Spanish origin were included in this study.

### DNA isolation and Quantification

Genomic DNA was isolated from peripheral blood lymphocytes by the salting-out method [33] and from saliva with Oragen DNA Self-Collection kit according to the manufacturer's recommendations (Genotek Inc. Ottawa, Ontario, Canada). DNA concentrations of all samples were determined on a NanoDrop spectrophotometer (NanoDrop Technologies, LLC, Wilmington, DE).

### Selection of genes and SNPs

We selected 58 functional candidate genes. Most belonged to gene pathways involved in the early paraxial mesoderm development, leading to formation of the occipital somites, such as retinoic acid (*RBP4*, *RBP1*, *STRA6*, *ADH4*, *RDH10*, *CRABP1*, *CYP26A1*, *CYP26C1*, *RARG*, *RARA*, *RXRA*, *ALDH1A2*) reviewed in [34], FGF8 (*FGF8*, *FGFR1*, *DUSP4*, *HES7*, *SNAI1*, *SNAI2*, *CDH1*) [35], [36], [37], Wnt (*WNT3A*, *CTNNB1*, *Axin2*, *T*, *TBX6*, *MSGN1*, *NKD1*, *NKD2*) [38], [39], [40], Notch (*DLL1*, *RBPJ*, *LFNG*, *NOTCH1*, *MESP2*, *ID1*, *ID2*) [37], [40], bone morphogenetic proteins (*BMPR1A*, *BMP2*) [41], Cdx (*CDX1*) [39], [42] and Hox pathways (*HOXA1*, *HOXA2*, *HOXA3*, *HOXA4*, *HOXB1*, *HOXB2*, *HOXB3*, *HOXB4*, *HOXC4*, *HOXD1*, *HOXD3*, *HOXD4*) reviewed in [42]. In addition, genes involved in sclerotome development, chondrogenesis and osteogenesis (*NOG*, *SHH*, *NKX3-2*, *PRRX1*) [43], [44], [45], [46], genes from the VEFG family crucial for placental angiogenesis (*VEGFA*, *KDR*, *FLT1*) [47], [48] and genes responsible for two conditions frequently associated with CMI, *NF1* (neurofibromatosis) and *STAT3* (Hyper-IgE Syndrome), were also included [49], [50].

The SNP selection was based on genetic coverage criteria, by considering linkage disequilibrium (LD) patterns within the candidate genes [51]. SNPs covering each gene plus 5 Kb flanking sequences were picked from the CEU panel of the HapMap database (www.hapmap.org, phases 1+2+3, release 27) [52], [53]. We used the LD-select software (droog.gs.washington.edu/ldSelect.html) to evaluate LD of the genomic regions in order to minimize redundancy between the selected SNPs [54], [55]. TagSNPs were selected with the following criteria: r^2^<0.85 from any other SNP according to CEU HapMap data and a minor allele frequency (MAF)>0.10 for genes with less than 20 tag SNPs and MAF>0.25 for those genes with more than 20 tagSNPs (*FLT1*, *KDR*, *NOTCH1*, *RXRA*).

### Plex design, genotyping and quality control

A total of 384 tagSNPs were initially selected in our study (Table S1): 77 SNPs for the retinoic acid pathway, 44 SNPs for the FGF8 pathway, 55 SNPs for the Wnt pathway, 44 SNPs for the Notch pathway, 24 SNPs for BMP, 6 SNPs for *CDX*, 38 SNPs for Hox pathways, 30 SNPs for genes involved in sclerotome development, 51 SNPs for the VEGF pathway and 15 SNPs for *NF1* and *STAT3*.

Genotyping was performed at the Barcelona node of the National Genotyping Center (CeGen, www.cegen.org) using the VeraCode technology (Illumina, San Diego, CA, USA) [56]. A total of 9 HapMap individuals were included as controls in the genotyping assay, obtaining a 100% concordance rate with HapMap genotype data. In addition, no differences were found in the genotypes of thirteen replicates.

### Statistical analysis

The analysis of minimal statistical power was performed *a priori* with the CaTS Power Calculator software (sph.umich.edu/csg/abecasis/CaTS) [57], assuming an odds ratio (OR) of 1.5, estimated disease prevalence of 0.008 [7], [10], [24], significance level of 0.05 (α) and MAF of 0.10, under the additive model.

All individuals with genotyping rates under 80% were excluded from the study. From 384 SNPs genotyped in our study, 303 passed quality controls filters and were included in the analysis, whereas 81 (21%) were excluded for the following reasons: more than 15% missing genotypes, deviation from Hardy-Weinberg equilibrium (HWE threshold set at p = 0.01 in our control population), monomorphism, MAF<0.10 or MAF<0.25 and *r^2^*>0.85 from any other studied SNP in our control sample. The evaluation of LD patterns and *r^2^* were performed from the genotype data of controls using Haploview v4.2 [58].

#### Single-marker analysis

The analysis of HWE and the case-control association study were performed with the SNPassoc R package [59]. For the case-control study we analysed all single markers under the additive model using the Cochran-Armitage Trend Test (ATT) (Table S1). A quantile-quantile plot (Q-Q plot) was generated with the ggplot2 R library [60]. The correction for multiple testing was performed using the Q-value R library assuming a 10% False Discovery Rate (FDR) [61] and the Bonferroni correction.

#### Multiple-marker analysis

To minimize multiple testing and type I (α) errors, we restricted the haplotype-based association study to only those LD blocks that included the SNPs overcoming the 10% FDR correction.

The linkage disequilibrium patterns for *CDX1*, *ALDH1A2* and *FLT1* were constructed from the genotyping data of our control individuals, using Haploview v4.2 software [58]. The haplotype-based analysis was performed using the same software [58]. To obtain a measure of significance for multiple testing in haplotype analysis, a total of 10,000 permutations were performed. Haplotypes with frequencies <0.05 were excluded.

#### PCF morphometric traits and risk genotypes

We used the Scheffé's post hoc test to evaluate whether common variants contributed to variation in any specific parameters as measured in PCF morphometric analyses, including cerebellar tonsillar descent, length of supraocciput, length of the clivus, foramen magnum diameter, PCF area, osseous PCF area, antero-posterior diameter of the PCF (PCF length), tentorium angle, basal angulation and Wackenheim angle, as previously described [32]. The analysis was carried out only in the 186 patients where the PCF morphometry confirmed the diagnosis of classical CMI and was limited to markers overcoming 10% FDR corrections for multiple testing in the single-marker analysis. The Kruskal-Wallis nonparametric test was used for variables not normally distributed (Kolmogorov-Smirnov test *P*<0.05) or showing no homogeneity of variances (Levene's test *P*<0.05)).

## Results

The most common signs and symptoms in the 415 cases included in the study are listed in Table 1. Headache was the most prevalent symptom (69.1%), often featuring neck irradiation (43.3% of those with headache), followed by dizziness (35.1%), motor weakness (33.6%), instability (32.1%), upper limb paresthesias (30.7%), sensory loss (29.7%) and fatigue (28.1%). Syringomyelia was present in 35.1% of cases. Hydrocephalus (15.1%) required specific management. Other, less common manifestations, as well as co-morbid conditions, were typical for CMI (Table 1). Positive CMI family history, defined as at least one MRI-confirmed CMI first relative, was present in only 6% of the cases. Surgical PCF reconstruction was performed in 55.8% of patients.

**Table 1:** Clinical findings in 415 patients with Chiari malformation type I.

	CMI
**Number of patients**	415
**Sex (M/F)**	122/293
**Age (y)**	42.2±50.9
**Age at diagnosis**	30.6±58.7/323
**Hydrocephalus**	57/377 (15.1%)
**Syringomyelia**	133/379 (35.1%)
**Intracranial Hypertension**	20/376 (5.3%)
**Retrocurved Odontoid**	40/352 (11.4%)
**Basilar impression**	11/382 (2.3%)
**Platibasia**	8/382 (2.1%)
**Klippel-Feil Malformation**	3/377 (0.8%)
**Other Craniocervical Malformations**	24/377 (6.4%)
**SIGNS AND SYMPTOMS**	
**Time elapsed from onset (mo)**	113.3±59.4/323
**Headache**	246/356 (69.1%)
**Occipital Headache/Cervicalgia**	97/224 (43.3%)
**Cough Headache**	87/358 (24.3%)
**Dizziness**	128/365 (35.1%)
**Vertigo**	82/365 (22.5%)
**Visual Alterations**	71/364 (19.5%)
**Nystagmus**	41/276 (14.8%)
**Kyphoscoliosis**	30/160 (18.7%)
**Fatigue**	102/363 (28.1%)
**Instability**	117/364 (32.1%)
**Sensory Loss**	108/364 (29.7%)
**Motor Weakness**	123/366 (33.6%)
**Dysphagia**	67/365 (18.3%)
**Dysphonia**	16/365 (4.4%)
**Gait Disturbances**	73/364 (20.0%)
**Paresthesia Upper Limbs**	112/365 (30.7%)
**Paresthesia Lower Limbs**	47/366 (12.9%)
**Upper Limb Pain**	47/365 (12.9%)
**Lower Limb Pain**	16/365 (4.4%)
**Neck Pain**	169/366 (46.2%)
**Difficulty Swallowing**	66/365 (18.1%)
**Anxiety**	38/360 (10.5%)
**Depression**	33/361 (9.1%)
**Neurofibromatosis type 1**	45/382 (11.8%)
**TREATMENT**	
**Posterior fossa reconstruction**	210/376 (55.8%)

Criteria for surgery were a >3 mm descent of the cerebellar tonsils in i) symptomatic patients, ii) oligosymptomatic patients with sleep apnea syndrome predominantly of the central type, and iii) patients with syringomyelia. Posterior fossa reconstruction was carried out in all patients to restore the volumetric capacity of the posterior fossa and to restore normal CSF dynamics at the craniovertebral junction. In summary, the technique consisted of the removal of a large amount of occipital bone and the posterior arch of the atlas. The dura mater was opened and in most cases an extra-arachnoidal technique was used. An extensive dural graft secured with tenting sutures to the cervical fascia was used to avoid arachnoid scarring and to allow for the expansion of the compressed cisterna magna or the creation of a pseudocisterna magna

Initially, 384 SNPs from 58 candidate genes were genotyped (Table S1). Eighty-one SNPs were excluded from statistical analysis after data filtering: 40 SNPs had a genotyping rate below 85%, 4 SNPs showed deviation from HWE, 20 SNPs were monomorphic or had MAF<0.10 (or MAF<0.25) and 17 had a r^2^>0.85 with other SNPs in the same candidate gene (Table S1).

All individuals with genotyping rates below 80% were excluded from the study, resulting in a range of genotyping efficiency of 94–97%. Thus, 303 SNPs were finally assessed in the association study in a total of 404 patients (286 female) and 519 sex-matched controls. The minimal statistical power of our sample, under the additive model, was 78% for the whole sample and 53% after subgrouping into classical CMI.

### Single Marker Analysis

We used a two-tiered single marker analysis. The first step comprised the whole patient sample while the second one included the more homogeneous subset of morphometry-proven classical CMI patients. In both we used the same control population.

**Figure 2:**
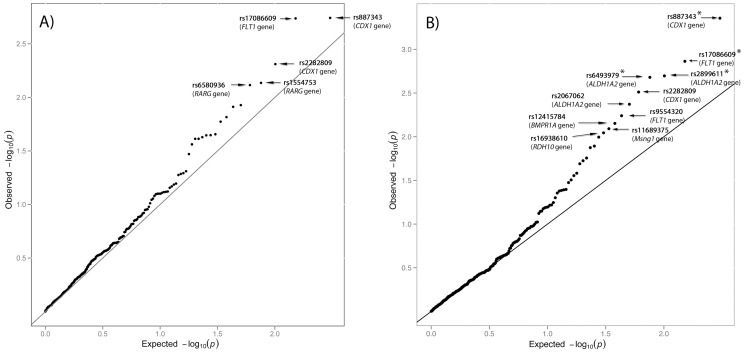
Quantile-quantile plot of the 303 *P*-values obtained in the association study under the additive model. 404 CMI patients versus 519 controls (A), and 186 small-PCF CMI versus 519 controls (B). SNPs with *P*-value<0.01 are indicated. Asterisks denote SNPs displaying association after 10% FDR correction for multiple testing (*P*<2.09E-03).

The case-control association study with the whole CMI sample (404 patients and 519 controls) found nominal associations (*P*-values<0.05) for 18 SNPs located in 14 genes (Table S1). Only 5 SNPs, located in three different genes, displayed *P*-values<0.01: *CDX1* (rs887343, rs2282809), *FLT1* (rs17086609) and *RARG* (rs1554753, rs6580936) (*P*-values and genotype counts are shown in Table 2 and Figure 2A, and *P*-values for the remaining SNPs are shown in Table S1). However, no marker remained significant after applying 10% FDR or the Bonferroni correction (*P* value = 1.6E-04 ( = 0.05/303 SNPs)).

**Table 2:** Case-control association study of 404 Spanish CMI patients and 519 sex-matched controls.

Gene		Control Genotypes N (%)			All CMI Genotypes N (%)				Classical CMI Genotypes N (%)			
	SNP (*P*<0.01)	11	12	22	11	12	22	*P*-val	11	12	22	*P*-val
***ALDH1A2***	rs2899611	110 (21)	264 (51)	145 (28)				NS	59 (32)	90 (48)	37 (20)	**0.00201** *
	rs6493979	78 (15)	273 (53)	168 (32)	84 (21)	207 (51)	113 (28)	0.02436	46 (25)	95 (51)	45 (24)	**0.00209** *
	rs2067062	148 (29)	258(50)	105 (21)				NS	35 (20)	93 (52)	51 (28)	**0.00425**
***BMPR1A***	rs12415784	5 (1)	135 (26)	379 (73)	5 (1)	76 (19)	323 (80)	0.02744	2 (1)	29 (16)	155 (83)	**0.00704**
***CDX1***	rs887343	85 (16)	252 (49)	180 (35)	43 (11)	187 (46)	174 (43)	**0.00181**	16 (9)	82 (44)	88 (47)	**0.00044** *
	rs2282809	71 (14)	240 (46)	206 (40)	37 (9)	174 (43)	193 (48)	**0.00489**	15 (8)	76 (41)	95 (51)	**0.00308**
***FLT1***	rs17086609	242 (47)	222 (43)	50 (10)	149 (37)	196 (49)	55 (14)	**0.00183**	67 (37)	83 (45)	33 (18)	**0.00137** *
	rs9554320	104 (20)	261 (50)	153 (30)				NS	25 (14)	88 (47)	73 (39)	**0.00575**
***MSGN1***	rs11689375	117 (23)	245 (47)	156 (30)	60 (15)	203 (51)	137 (34)	0.01225	21 (11)	98 (53)	64 (36)	**0.00810**
***RARG***	rs1554753	310 (60)	182 (35)	27 (5)	272 (67)	121 (30)	11 (3)	**0.00732**	146 (79)	36 (19)	4 (2)	0.01752
	rs6580936	358 (69)	146 (28)	15 (3)	310 (77)	87 (21)	7 (2)	**0.00767**				NS
***RDH10***	rs16938610	305 (59)	178 (34)	36 (7)	262 (64)	126 (31)	16 (4)	0.02209	127 (68)	53 (29)	6 (3)	**0.00903**

An additional study analyzed 186 classical CMI patients and the same control population. Only markers with *P*-value<0.01 (Cochran-Armitage trend test) are shown.

* Significant associations after applying 10% FDR.

The second analysis considered 186 classical CMI patients (patients with MRI morphometry-confirmed small PCF) and the 519 controls. Nominal associations (*P*-values<0.05) were found under the additive model for 26 SNPs located in 13 genes (Table S1). The following 10 SNPs, located in 6 different genes, displayed *P*-values<0.01: *ALDH1A2* (rs2899611, rs6493979, rs2067062), *BMPR1A* (rs12415784), *CDX1* (rs887343, rs2282809), *FLT1* (rs17086609, rs9554320), *MSGN1* (rs11689375) and *RDH10* (rs16938610) (Table 2 and Figure 2B). *P-*values for the remaining SNPs are shown in Table S1.

After applying a 10% FDR correction (*P*<2.09E-03) four associations did remain significant: rs887343 (*P* = 4.4E-04) in *CDX1*, rs17086609 (*P* = 1.37E-03) in *FLT1*, rs2899611 (*P* = 2.01E-03) and rs6493979 (*P* = 2.09E-03) in the *ALDH1A2* gene. Under the more strict Bonferroni correction no SNP reached significance.

### Multiple Marker Analysis

**Figure 3:**
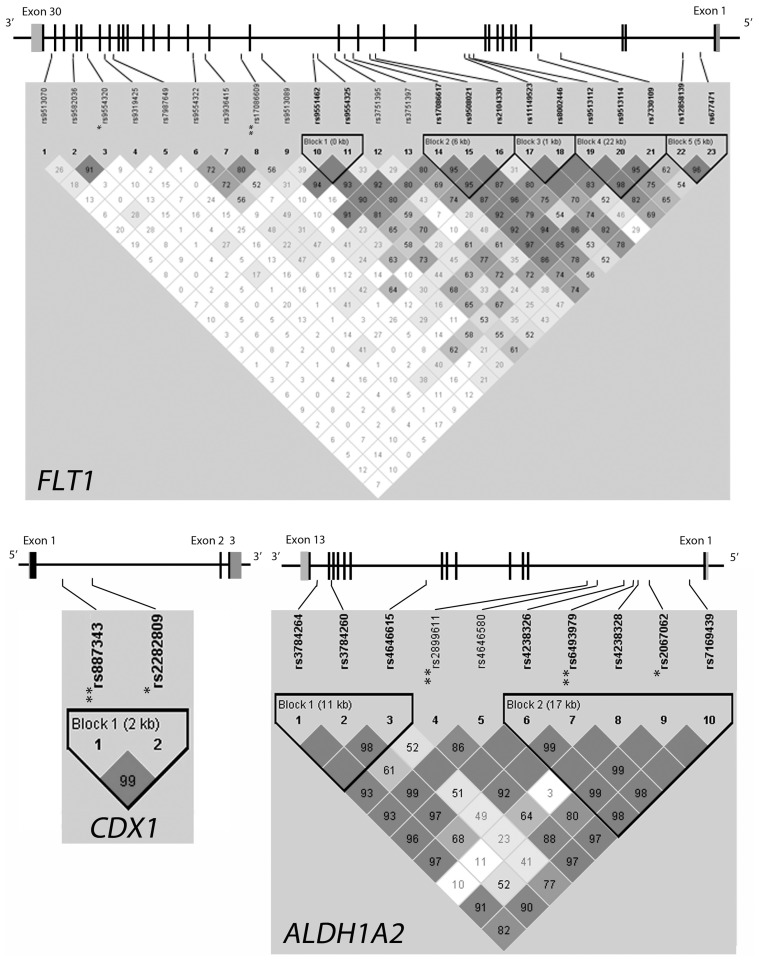
Haploview graphs showing the markers tested and haplotype blocks constructed for *ALDH1A2*, *CDX1* and *FLT1*. D′ values are indicated (tones from white to dark grey indicate D′ values from 0 to 1, respectively). The genomic structures of *ALDH1A2*, *CDX1* and *FLT1* genes are drawn with coding exons indicated as black boxes. The SNPs with *P*-value<0.01 are indicated with an asterisk (*); the SNPs showing association after 10% FDR correction for multiple testing (*P*<2.09E-03) are indicated with a double asterisk (**).

The haplotype analysis was addressed only for those genes containing SNPs that passed 10% FDR in the single marker analysis. The pairwise linkage disequilibrium map of *ALDH1A2*, *CDX1* and *FLT1* genes was calculated in the sample under study (Figure 3). LD patterns across the *FLT1* gene defined five blocks (block 1 of <1 kb: rs9551462, rs9554325; block 2 of 6 kb: rs17086617, rs9508021 and rs2104330; block 3 of 1 kb: rs11149523 and rs8002446; block 4 of 22 kb: rs9513112, rs9513114 and rs7330109; block5 of 5 kb: rs12858139 and rs677471); two blocks were defined in *ALDH1A2* (block 1 of 11 kb: rs3784264, rs3784260, rs4646615; block 2 of 17 kb: rs4238326, rs6493979, rs4238328, rs2067062 and rs7169439) and only one block in *CDX1* (block 1 of 2 kb: rs887343 and rs2282809). The haplotype analysis was assessed only for those blocks containing SNPs significantly associated (block2 of *ALDH1A2* and the only block of *CDX1)* (Figure 3). The SNPs rs2899611 (in *ALDH1A2*) and rs17086609 (in *FLT1*) were singletons (Figure 3). The results in the *CDX1* gene identified a risk haplotype constituted of G-G allelic combination (Permutated *P*-value = 9.4 E-03; OR = 1.54, 95% CI = 1.19–2.01) (Table 3). The results in the *ALDH1A2* gene identified an over-representation of the C-A-G-C-G haplotype in cases (Permuted *P*-value = 0.0179; OR = 1.53, 95% CI = 1.16–2.02).

**Table 3:** Haplotype analysis for the linkage disequilibrium blocks containing SNPs that showed significant associations in the single marker analysis (Haploview v4.2 software).

Population	Gene	LD block: SNPs	Haplotype	Cases %	Controls %	*P*-value (permP)^a^	Odds Ratio [95% CI]
Classical CMI	*CDX1*	Block 1: rs887343-rs2282809	GG	255 (71)	614 (62)	0.0014 (0.0094)	1.54 [1.19–2.01]
			CA	103 (29)	384 (38)	0.0012 (0.0092)	0.65 [0.50–0.84]
Classical CMI	*ALDH1A2*	Block 2: rs4238326-rs6493979-rs4238328-rs2067062-rs7169439	TGGAG	99 (28)	328 (33)	0.0732 (0.3975)	0.79 [0.60–1.02]
			CAGCG	100 (28)	201 (20)	0.0025 (0.0179)	1.53 [1.16–2.02]
			TGAAG	66 (19)	233 (24)	0.0563 (0.3233)	0.75 [0.55–1.01]
			CAGCA	49 (14)	140 (14)	0.882 (1.0000)	0.97 [0.69–1.38]
			TAGCG	38 (11)	87 (9)	0.2624 (0.8997)	1.24 [0.83–1.86]

a A total of 10,000 permutations were performed using Haploview v4.2 to obtain a measure of significance corrected for multiple testing.

### PCF morphometric traits and risk genotypes

We sought to identify a possible relationship between genotypes of the four SNPs in three genes that passed 10% FDR correction (rs289961, rs6493979 (*ALDH1A2*), rs887343 (*CDX1*), rs17086609 (*FLT1*)) and any of the PCF morphological measurements performed on the MRI studies of 211 patients (Table 4). A modest association was found between the two SNPs in *ALDH1A2* and the Wackenheim angle and the degree of basal angulation (*P*-value<0.05).

**Table 4:** Association between risk genotypes and PCF morphometric traits in 211 patients with CMI.

	*ALDH1A2*	*ALDH1A2*	*CDX1*	*FLT1*
Measures	rs2899611	rs6493979	rs887343	rs17086609
**TD**	0.105	0.684	0.226	0.328
**Supraocciput**	0.477	0.352	0.777	0.829
**Clivus**	0.922	0.976	0.593	0.769
**FM**	0.080	0.595	0.227	0.933
**PCF area**	0.248	0.560	0.556	0.459
**OPCF area**	0.367	0.636	0.532	0.356
**PCF length** *	0.750	0.300	0.094	0.983
**Tentorium angle**	0.456	0.836	0.410	0.118
**Basal angulation**	**0.016**	0.067	0.800	0.228
**Wackenheim angle**	**0.006**	**0.039**	0.824	0.146

Data presented are *P*-values (Scheffé's post-hoc and Kruskal-Wallis tests).

* Assessed as the distance from the dorsum sellae to the internal occipital protuberance; in bold, P<0.05.TD: tonsillar descent; OPCF: osseous PCF, FM: foramen magnum.

## Discussion

In the present association study performed in a sample of CMI patients from the Spanish population we found nominal association for SNPs in several genes involved in the early development of paraxial mesoderm and vascular development of the placenta. After corrections for multiple testing, only associations with four SNPs within *CDX1*, *FLT1* and *ALDH1A2* remained significant in the subsample where MRI morphometric analysis documented a reduced PCF. Considering these four SNPs, we also found a modest correlation between the two in *ALDH1A2* and specific morphometric data of the PCF.

The Chiari malformations have long been considered sporadic conditions, without a heritable etiology. However, there have been a number of case reports identifying familial aggregation and clustering of CMI, suggesting a genetic basis [9], [25] although the involved genes and even type of inheritance are presently unknown. Given the predominance of apparently sporadic patients and the relatively high prevalence of the disorder, a polygenic model of inheritance seems plausible in a majority of CMI cases. To our knowledge, however, this is the first genetic association study ever to be carried out in CMI.

The contribution of common genetic variants has been examined in Chiari malformation type II (CMII), where several studies failed to demonstrate consistent associations with developmental genes such as *CRABP1*, *CRABP2*, *ALDH1A2*, *RALDH2*, *CYP26*, *HOX*, *NOG*, *SHH* and *TBX6* (reviewed in [62], [63], [64]). Mutational screens of some of these genes did not find definitive evidence for disease-causing mutations [63]. Since the most accepted pathogenic hypothesis in CMI is that of insufficiency of paraxial mesoderm in the third and fourth week of gestation, we decided to explore whether variants in genes expressed during somitogenesis could be associated with the milder and more frequent CMI phenotype.

### Association study results

A number of nominal associations were found in the whole CMI sample. Despite none of them overcoming corrections for multiple testing, three of the selected candidate genes, *CDX1*, *FLT1* and *RARG*, contained SNPs that showed association with *P*-values<0.01, thus suggesting that these genes are worth of scrutiny in further samples. However, our cohort was selected according to the radiographic standard criterion for diagnosis of CMI, a cerebellar tonsillar descent exceeding 3 mm on midsagittal MRI. Application of this single criterion may have resulted in clinical (and presumably genetic) heterogeneity, as in fact is suggested by the PCF morphometric data we obtained in the subgroup of cases where digitized MRI studies were available. Thus, it is not surprising that statistical significance of the association was higher in the group of 186 patients with underdeveloped PCF. Among the 26 nominally associated SNPs within 13 genes, in this “classical CMI” subsample, association with *P*-values<0.01 were found for SNPs within six genes: *ALDH1A2*, *RDH10*, *CDX1*, *BMPR1A*, *MSGN1* and *FLT1*. The association remained significant after a 10% FDR correction for SNPs within three genes: rs887343 in *CDX1*, rs17086609 in *FLT1* and rs2899611 plus rs6493979 in the *ALDH1A2* gene.

Regarding the potential functional relevance of the identified variants, four of the genes with the best association signals (*P*<0.01 in the single marker analysis of either CMI or classical CMI), *ALDH1A2*, *RDH10*, *RARG* and *CDX1*, are directly or indirectly related with retinoic acid (RA) signalling during somitogenesis. RA is a transcription factor needed for elongation of the embryonic body axis, acting as a molecular oscillator in the somitic precursors of the paraxial mesoderm [39] and promoting somite differentiation through regulation of the Hox genes [42]. Expression of RA synthetic enzyme, RALDH2, encoded by *ALDH1A2*, is crucial at these presomitic or early somatic stages. Avian or mouse RA-deficient embryos develop abnormally small somites (reviewed in [34]), while exposure to teratogenic RA levels give rise to congenital dysraphism in humans [65]. *RDH10* encodes a retinol dehydrogenase critical for embryonic vitamin A metabolism. Murine *Rdh10* mutants display lethal abnormalities characteristic of a RA-deficiency phenotype [66]. RARG binds to DNA after heterodimerization with any of the retionid X receptors, leading to activation of RA response elements (RAREs), which displace transcriptional corepressors and recruits coactivators [67]. Through this pathway, RA regulates specific Hox expression in paraxial mesoderm, determining embryonic anterior-posterior patterning throughout somitogenesis (reviewed in [34], [67]). This may origin spinal column defects such as scoliosis [68], present in nearly 20% of our CMI patients. A third RA-related gene showing association is *CDX1*. *Cdx* genes encode homeodomain transcription factors containing atypical RARE [69], [70]. In the mouse embryo primitive streak, expression of *Cdx1* begins at postconceptional day 7.5 in concurrence with onset of RA signalling and Hox expression. Of note, *Cdx1*-null mutants display alterations in the basioccipital bone [69].

The other three listed genes, *BMPR1A*, *MSGN1* and *FLT1*, belong to less interrelated pathways. *BMPR1A* has been found to regulate the recruitment of prospective paraxial mesoderm cells in the mouse epiblast. Mutant embryos have a shorter primitive streak, a more proximal node and a defective extension of the primitive streak [41]. *MSGN1* is a direct target of *Wnt* and *TBX6* during the specification, maturation and segmentation of the paraxial mesoderm in the mouse [38]. *FLT-1* encodes VEGFR-1, a vascular endothelial growth factor receptor expressed in the endothelium of blood vessels [71]. Vasculogenesis at the chorionic villi tree is evident by post conceptional day 21, during the four-somite embryonic stage (reviewed in [72]). A *FLT-1* mutation resulted in endothelial disorganization and abnormal vessel formation during early embryo development [73]. During the fourth week of life the human embryo switches from the exchange of nutrients and wastes by simple diffusion to establishment of utero-placental circulation. It is conceivable that failure to properly transition through these two stages could impair critical processes as mesodermal proliferation or neurulation.

Finally, the four variants associated with classical CMI (rs887343 in *CDX1*, rs17086609 in *FLT1* and rs2899611 plus rs6493979 in *ALDH1A2*) are intronic, with no direct effect on the protein sequence. It could be that the identified risk variants may produce by themselves functional alterations or they might lie in LD with other susceptibility loci, in the case of an indirect association. Since all these SNPs are tagSNPs, we analyzed these variants and those in LD with them by means of the web utility SNP Function Prediction [74]. The predictions of functional effects for transcription factor binding sites, exonic splicing enhancers or silencers, changing of splicing pattern or efficiency by disrupting splice sites, regulation of protein translation by affecting microRNA binding sites and conservation scores were all negative.

### Genetic-morphometric analysis

We also investigated whether any specific PCF morphological feature might relate to any of the gene variants showing association with CMI. A short anteroposterior diameter of the PCF and an increased basal angle have been described in some CMI morphometric studies [17], [19]. However, no correlation was found among genotypes and any of the previously considered more typical features of CMI, such as clivus or supraocciput length, PCF area or tentorial angle [15]. In our study, two SNPs in *ALDH1A2* displayed a correlation with the Wackenheim angle and one of them with the basal angulation, in keeping with previous evidence pointing at basiocciput, rather than supraocciput, abnormalities in CMI. Whether an abnormal clivus slope does constitute a marker of genetically-determined CMI deserves further investigation.

### Methodological issues

The present case–control association study raises several methodological questions. First, the modest sample size (404 patients and 519 controls) may have prevented from detecting subtle phenotypic effects, as the statistical power calculated for this cohort is close to 80% when an OR of 1.5 is assumed. Second, we do not present a replication study of our findings in an independent cohort. This notwithstanding, ours was a relatively large sample considering the low prevalence of CMI (1∶1000 to 1∶5000), bordering that of rare diseases. It was also the first CMI sample being explored for association with a large set of candidate genes. Third, although the SNP selection was designed to cover the 58 candidate genes on the basis of existing linkage disequilibrium patterns, gaps still exist in 12 genes due to experimental constraints, and 21% of the 384 SNPs could not be studied. Last, lack of MRI studiy of the control population may have resulted in inclusion of some asymptomatic CMI cases, although given the estimated low prevalence of tonsillar descent, this should not be expected to influence the validity of our findings

In conclusion our genetic data give support to the hypothesis that variants in genes involved in paraxial mesoderm may determine PCF size to the extent of the development of the CMI. Specifically, our results point to a putative role for defective RA signalling and fetal vasculogenesis in causing hypoplasia of the basichondrocranium. Although confirmatory studies are needed, these are among the first genetic susceptibility factors to be defined in CMI. Given the limitations of the current radiographic diagnostic criteria, understanding of the genetic underpinnings of CMI should have important clinical implications, including earlier recognition of subjects at risk of becoming symptomatic and the timely indication of the surgical reconstruction of the PCF.

## Supporting Information

Table S1 Description of the 384 SNPs initially selected for the genotyping assay using the VeraCode technology.
(DOCX)
