# Supplementary material for: Chiari Malformation Type I: A Case-Control Association Study of 58 Developmental Genes
Source: PLoS One. 2013 Feb 21;8(2):e57241. doi: 10.1371/journal.pone.0057241 (PMC3578784; doi:10.1371/journal.pone.0057241)
Supplement: Table S1 — Description of the 384 SNPs initially selected for the genotyping assay using the VeraCode technology. (DOCX) [file pone.0057241.s001.docx]

| **Table S1.** Description of the 384 SNPs initially selected for the genotyping assay using the VeraCode Technology. | | | | | | |  |  |  |
| --- | --- | --- | --- | --- | --- | --- | --- | --- | --- |
| **Gene (cov**^a^**)** | | **Location** | **TagSNP_ID** | **SNPs captured**^b^ | **Genotype Call Rate (%)** | **MAF**^c^ | **HWE^d^**  ***(p-val*)** | **Case/Control (*p-val*)**^e^  **All patients** | **Case/Control (*p-val*)**^e^ **Classical CMI** |
| *ADH4* (1) | | 4q22 | rs17218560^1^ | rs2602880, rs2602880, rs2602883, rs2851255, rs2602891, rs2851253, rs2924583, rs1042365, rs1042364, rs2602895, rs2851248, rs2851247, rs2851246, rs2602896, rs2602878, rs34936974, rs13129488, rs29001203, rs17817359, rs17217949, rs17218003, rs13143133, rs17218073, rs17218108, rs13112176, rs17218141, rs17218162, rs17218239, rs17218288, rs13133647, rs7669636, rs7670060, rs7670241, rs7694646, rs4699712, rs4699713, rs4699714, rs17817868, rs1126672, rs17218602, rs17817958, rs13110176, rs13110764, rs13138294, rs2602877, rs6532795, rs6532796, rs1126673, rs1126671, rs6837685, rs13113166, rs1126670, rs6532798, rs7689753, rs10017466, rs7670638, rs3919370, rs4699716, rs4699717 |  |  |  |  |  |
|  |  |  | rs1800759 | - | 100 | 0.42 | 0.72 | 0.401 | 0.75075 |
|  |  |  | rs2602884 | - | 99.9 | 0.15 | 0.29 | 0.757 | 0.86450 |
|  |  |  | rs3762894 | - | 99.8 | 0.18 | 1 | 0.345 | 0.36130 |
|  |  |  | rs3828541^4^ | rs10009145, rs13148577 |  |  |  |  |  |
|  |  |  | rs4148883 | - | 98.8 | 0.48 | 0.42 | 0.310 | 0.98474 |
|  |  |  | rs4699710 | - | 100 | 0.33 | 0.62 | 0.709 | 0.51922 |
| *ALDH1A2* (0.85) | | 15q21.3 | rs2067062 | rs2704218 | 97 | 0.48 | 0.72 | 0.051 | 0.00425 |
|  |  |  | rs2899611 | - | 100 |  | 0.66 | 0.077 | 0.00201 |
|  |  |  | rs3784260 | rs10851631, rs12907038, rs7495968, rs9325, rs3204689, rs4646640, rs12910113, rs4646638, rs11855259, rs4646636, rs7165247, rs3784263, rs3784262, rs10851630, rs4646616, rs12903551, rs12903474, rs12908953, rs12915901, rs12148907, rs12901462 | 100 | 0.46 | 0.86 | 0.229 | 0.07091 |
|  |  |  | rs3784264 | rs8041644, rs1441815, rs2642630 | 97.2 | 0.41 | 0.31 | 0.460 | 0.02039 |
|  |  |  | rs4238326 | rs11071365, rs4646576, rs4646572, rs4646568 | 99.8 | 0.35 | 0.06 | 0.049 | 0.01333 |
|  |  |  | rs4238328 | - | 99.9 | 0.21 | 0.90 | 0.153 | 0.05668 |
|  |  |  | rs4646580 | rs10851632, rs1441819, rs4646645, rs1061278, rs1063666, rs3204690, rs4646642, rs2197092, rs3742961, rs3742960, rs3742959, rs4646626, rs4646625, rs2414527, rs3784259, rs3784257, rs12594082, rs4646609, rs12898976, rs8026952, rs12907125, rs12914388, rs1822205, rs4646607, rs1899355, rs4646606, rs4646605, rs7180059, rs12917536, rs1441829, rs12909423, rs1372369, rs4646600, rs4646595, rs4646594, rs4646590, rs4646589, rs4646588, rs4646584, rs4646582, rs4646581, rs2642632, rs7178598, rs12903724, rs12148281, rs8032572, rs4646579, rs12595180, rs12912093, rs2899613, rs1994927, rs17820990, rs12914603, rs4646564, rs6493981, rs2704225, rs4646560, rs11071367, rs4646559, rs4646555, rs4646554, rs2414530, rs7171261 | 99.9 | 0.45 | 0.79 | 0.180 | 0.01280 |
|  |  |  | rs4646615 | - | 99.2 | 0.35 | 0.63 | 0.633 | 0.40794 |
|  |  |  | rs6493979 | rs11071364, rs10518961, rs1994926, rs8027188, rs4238325, rs4238327, rs17820966, rs4144005 | 100 | 0.44 | 0.06 | 0.024 | 0.00209 |
|  |  |  | rs7169289^4^ | - |  |  |  |  |  |
|  |  |  | rs7169439 | - | 99.9 | 0.13 | 0.26 | 0.659 | 0.83777 |
| *Axin2* (1) | | 17q23-q24 | rs11079571 | - | 100 | 0.22 | 0.52 | 0.831 | 0.17468 |
|  |  |  | rs11655966 | - | 99.6 | 0.24 | 0.71 | 0.580 | 0.75120 |
|  |  |  | rs11868547 | - | 100 | 0.41 | 0.86 | 0.299 | 0.15239 |
|  |  |  | rs12452196 | - | 100 | 0.17 | 0.54 | 0.760 | 0.61896 |
|  |  |  | rs2240308^4^ | - |  |  |  |  |  |
|  |  |  | rs3923086 | - | 100 | 0.50 | 0.86 | 0.920 | 0.87306 |
|  |  |  | rs3923087 | - | 99.9 | 0.30 | 0.91 | 0.458 | 0.22702 |
|  |  |  | rs4074947 | - | 99.8 | 0.25 | 0.90 | 0.498 | 0.12799 |
|  |  |  | rs4541111^4^ | - |  |  |  |  |  |
|  |  |  | rs4791171 | rs11867417 | 100 | 0.34 | 0.54 | 0.120 | 0.22860 |
|  |  |  | rs7224837^3^ | rs7210356 |  |  |  |  |  |
|  |  |  | rs740026 | - | 99.9 | 0.39 | 0.46 | 0.820 | 0.96824 |
|  |  |  | rs757558 | - | 99.9 | 0.17 | 0.54 | 0.540 | 0.32799 |
|  |  |  | rs7591 | - | 99.9 | 0.41 | 0.93 | 0.298 | 0.11181 |
| *NKX3-2* (1) | | 4p16.3 | rs10939485^4^ | - |  |  |  |  |  |
|  |  |  | rs16888802^4^ | - |  |  |  |  |  |
|  |  |  | rs3822226^1^ | - |  |  |  |  |  |
|  |  |  | rs758974 | rs758973, rs12501307, rs1476211 | 100 | 0.17 | 0.19 | 0.070 | 0.44272 |
| *BMP2* (1) | | 20p12 | rs1005464 | - | 100 | 0.20 | 0.34 | 0.705 | 0.36768 |
|  |  |  | rs170986 | - | 100 | 0.17 | 0.87 | 0.294 | 0.16656 |
|  |  |  | rs1979855 | - | 99.7 | 0.19 | 0.12 | 0.638 | 0.21651 |
|  |  |  | rs1980499 | - | 100 | 0.47 | 0.66 | 0.474 | 0.51044 |
|  |  |  | rs235764 | - | 99.9 | 0.31 | 0.47 | 0.678 | 0.47010 |
|  |  |  | rs235767 | - | 99.9 | 0.44 | 0.86 | 0.130 | 0.66840 |
|  |  |  | rs235770^1^ | rs6054512, rs235768, rs235772 |  |  |  |  |  |
|  |  |  | rs235771 | - | 100 | 0.36 | 0.57 | 0.624 | 0.61712 |
|  |  |  | rs28488^1^ | - |  |  |  |  |  |
|  |  |  | rs3178250 | - | 99.8 | 0.25 | 0.13 | 0.279 | 0.03122 |
|  |  |  | rs6117432 | - | 99.9 | 0.22 | 0.70 | 0.666 | 0.27882 |
| *BMPR1A* (1) | | 10q22.3 | rs10887666 | rs4934275, rs11202231, rs11202232, rs7895131, rs10788533, rs10887664, rs11594941, rs10788528, rs4934280, rs11594289, rs7094713 | 100 | 0.16 | 0.44 | 0.514 | 0.09989 |
|  |  |  | rs11202185 | - | 99.8 | 0.37 | 0.34 | 0.152 | 0.50827 |
|  |  |  | rs11202222^4^ | - |  |  |  |  |  |
|  |  |  | rs11594232 | - | 99.9 | 0.25 | 0.29 | 0.434 | 0.78817 |
|  |  |  | rs12415784 | - | 100 | 0.12 | 0.07 | 0.027 | 0.00704 |
|  |  |  | rs12765929 | rs17335915 | 100 | 0.27 | 0.07 | 0.111 | 0.01011 |
|  |  |  | rs2168730 | rs2125055, rs4934272, rs7095025, rs4481940, rs4933416, rs4933417, rs11202221 | 99.7 | 0.25 | 0.90 | 0.626 | 0.43565 |
|  |  |  | rs4934263 | - | 99.9 | 0.41 | 0.64 | 0.079 | 0.06040 |
|  |  |  | rs6586037 | rs7074582, rs2353786, rs6586034, rs6586035, rs7916903, rs3858284, rs4934262, rs4934265, rs2125054, rs4934268, rs7906823, rs2354353, rs2883420, rs7905807, rs4934279 | 95.1 | 0.43 | 0.41 | 0.307 | 0.30566 |
|  |  |  | rs7096781 | rs7095804, rs4934261, rs2168727, rs10887654, rs3905377, rs7088641 | 99.5 | 0.32 | 0.76 | 0.779 | 0.24694 |
|  |  |  | rs733877^3^ | - |  |  |  |  |  |
|  |  |  | rs7895217^1^ | rs1124482, rs4933411, rs10887656, rs2354352, rs11202197, rs4934269, rs4478900, rs7478254, rs4933415, rs4934273, rs10887658, rs2125057, rs722068, rs2354354, rs2354355, rs11202227, rs10887660, rs7087358, rs7091555 |  |  |  |  |  |
|  |  |  | rs7922846 | rs10749542, rs749673, rs7074064 | 98.8 | 0.29 | 0.02 | 0.273 | 0.30817 |
| *CDH1* (0.89) | | 16q22.1 | rs11075699 | - | 99.6 | 0.43 | 0.10 | 0.874 | 0.88966 |
|  |  |  | rs12185157^4^ | rs1078621, rs7203337, rs11642413, rs4783676, rs1125557, rs12448999 |  |  |  |  |  |
|  |  |  | rs12597188 | - | 100 | 0.31 | 0.07 | 0.443 | 0.52844 |
|  |  |  | rs16958383 | - | 100 | 0.14 | 0.58 | 0.700 | 0.44864 |
|  |  |  | rs17690554 | rs13689 | 100 | 0.15 | 0.39 | 0.881 | 0.42987 |
|  |  |  | rs2276330 | - | 99.9 | 0.12 | 0.07 | 0.320 | 0.18831 |
|  |  |  | rs3785078 | rs8061932 | 99.9 | 0.13 | 0.54 | 0.653 | 0.49525 |
|  |  |  | rs7186053 | rs10431923, rs10431924 | 100 | 0.40 | 0.10 | 0.360 | 0.37972 |
|  |  |  | rs7186084^1^ | - |  |  |  |  |  |
|  |  |  | rs7188750 | - | 100 | 0.17 | 1 | 0.863 | 0.37428 |
|  |  |  | rs7196495 | rs7196661, rs2902185, rs13335476, rs4783672, rs9941051, rs11640099, rs9940250, rs4783673, rs1974873, rs12931189, rs4783572 | 100 | 0.10 | 0.64 | 0.770 | 0.92258 |
|  |  |  | rs7203904 | - | 99.9 | 0.22 | 0.90 | 0.496 | 0.91403 |
|  |  |  | rs9925923^1^ | rs2059254, rs7186333, rs16260, rs11865026, rs7200690, rs17772363, rs13333528, rs9646284, rs9928847, rs13334471, rs8056538, rs12930371, rs12446407, rs12446413, rs12443730, rs4783681, rs4783570, rs13334326, rs13339591, rs9282650, rs17772411, rs2113199, rs12599517, rs2113200, rs2113201, rs7199991, rs7198799, rs2961, rs1981871, rs9929218, rs9929239, rs9929479, rs12919719, rs12924033, rs4076177, rs12599393, rs17715799, rs2010724, rs1075959, rs1862748, rs4783686 |  |  |  |  |  |
|  |  |  | rs9935563 | rs1801552 | 99.7 | 0.36 | 0.25 | 0.808 | 0.85441 |
| *CDX1* (0.83) | | 5q32 | rs2282809 | rs2282808, rs2282810, rs2282812 | 99.8 | 0.34 | 0.92 | 0.005 | 0.00308 |
|  |  |  | rs3776083^4^ | - |  |  |  |  |  |
|  |  |  | rs6890699^4^ | - |  |  |  |  |  |
|  |  |  | rs6894617^4^ | - |  |  |  |  |  |
|  |  |  | rs887343 | - | 99.8 | 0.38 | 0.93 | 0.002 | 0.00044 |
|  |  |  | rs887344^4^ | rs2302275, rs3776082, rs2237091, rs717746 |  |  |  |  |  |
| *CRABP1* (0.5) | | 15q24 | rs3813573 | rs2139440 | 100 | 0.20 | 0.69 | 0.269 | 0.88354 |
|  |  |  | rs8027180 | - | 99.8 | 0.5 | 0.33 | 0.090 | 0.82392 |
| *CTNNB1*(1) | | 3p21 | rs4135385 | - | 99.8 | 0.23 | 0.38 | 0.359 | 0.60125 |
|  |  |  | rs9311265 | rs3864004, rs4533622, rs2371452, rs3915129, rs6776881, rs2140090, rs4973927, rs13076290, rs1798802, rs13072632, rs1880481, rs11564450, rs11564452, rs2953, rs9870255, rs9883073, rs1722845, rs9813198, rs13075993, rs3774369, rs11564454, rs3774371, rs11564465, rs11129895 | 99.9 | 0.43 | 0.13 | 0.743 | 0.15597 |
| *CYP26A1* (1) | | 10q23-q24 | rs10882140^4^ | - |  |  |  |  |  |
|  |  |  | rs7905501 | rs17465590, rs7905939 | 99.8 | 0.20 | 1 | 0.891 | 0.39933 |
|  |  |  | rs11812460 | rs4411227 | 99.9 | 0.27 | 0.16 | 0.364 | 0.31404 |
| *CYP26C1* (1) | | 10q23.33 | rs12256889 | rs4919594 | 100 | 0.32 | 0.09 | 0.432 | 0.50127 |
|  |  |  | rs1339820 | - | 98.3 | 0.11 | 1 | 0.333 | 0.63270 |
|  |  |  | rs1538648 | - | 99.7 | 0.47 | 0.09 | 0.088 | 0.33212 |
|  |  |  | rs2068888 | rs4418728 | 99.9 | 0.47 | 1 | 0.873 | 0.79843 |
|  |  |  | rs7073161^4^ | - |  |  |  |  |  |
|  |  |  | rs7922067^4^ | rs8211 |  |  |  |  |  |
| *DLL1* (1) | | 6q27 | rs1028489 | rs1028488 | 99.8 | 0.49 | 0.22 | 0.766 | 0.85004 |
|  |  |  | rs1033583 | - | 99.9 | 0.34 | 0.55 | 0.197 | 0.24311 |
|  |  |  | rs2738820 | - | 99.9 | 0.35 | 0.49 | 0.142 | 0.16199 |
|  |  |  | rs9356632^4^ | rs2180052 |  |  |  |  |  |
|  |  |  | rs9460102 | - | 100 | 0.18 | 0.54 | 0.670 | 0.56402 |
|  |  |  | rs9460103 | - | 98.7 | 0.39 | 0.30 | 0.257 | 0.64103 |
| *DUSP4* (1) | | 8p12-p11 | rs12540995 | - | 99.7 | 0.25 | 0.91 | 0.469 | 0.70954 |
|  |  |  | rs2056025 | rs7831728, rs1455915, rs7823775 | 100 | 0.11 | 0.49 | 0.891 | 0.90819 |
|  |  |  | rs2341674 | - | 99.9 | 0.18 | 0.66 | 0.151 | 0.58428 |
|  |  |  | rs3824133^1^ | - |  |  |  |  |  |
|  |  |  | rs473004 | rs474824 | 100 | 0.36 | 1 | 0.765 | 0.73181 |
|  |  |  | rs567436 | rs569209, rs2637819 | 100 | 0.25 | 0.73 | 0.826 | 0.69406 |
| *FGF8* (1) | | 10q24 | rs1348870 | - | 99.9 | 0.39 | 0.85 | 0.999 | 0.64623 |
| *FGFR1* (1) | | 8p12 | rs10105686 | rs6474354 | 99.7 | 0.22 | 0.38 | 0.235 | 0.87953 |
|  |  |  | rs10958704 | - | 99.8 | 0.37 | 0.71 | 0.131 | 0.11048 |
|  |  |  | rs17182127^4^ | rs17182120, rs7825208 |  |  |  |  |  |
|  |  |  | rs2288696 | - | 99.8 | 0.20 | 0.90 | 0.197 | 0.60851 |
|  |  |  | rs2411256 | - | 100 | 0.21 | 0.58 | 0.079 | 0.48876 |
|  |  |  | rs2978076^3^ | rs2280846, rs2956724, rs2978073 |  |  |  |  |  |
|  |  |  | rs2978083 | - | 99.9 | 0.02 | 0.33 |  |  |
|  |  |  | rs3758102 | - | 99.8 | 0.22 | 0.68 | 0.017 | 0.06674 |
|  |  |  | rs3925^1^ | rs2411257, rs10958700, rs13317, rs3779921, rs2293971, rs2304000, rs4647907, rs10101096, rs16887378, rs11777067 |  |  |  |  |  |
|  |  |  | rs4733930 | - | 100 | 0.43 | 0.37 | 0.882 | 0.94066 |
|  |  |  | rs6983315 | rs6987534 | 100 | 0.41 | 0.47 | 0.477 | 0.48755 |
|  |  |  | rs6996321 | rs12677355 | 100 | 0.41 | 0.31 | 0.224 | 0.11976 |
|  |  |  | rs7012413 | - | 99.6 | 0.31 | 0.76 | 0.893 | 0.51959 |
| *FLT1* (1) | | 13q12 | rs10507385^3^ | - |  |  |  |  |  |
|  |  |  | rs11149523 | - | 99.5 | 0.32 | 0.70 | 0.173 | 0.23780 |
|  |  |  | rs12858139 | rs655024 | 99.8 | 0.42 | 0.07 | 0.401 | 0.43052 |
|  |  |  | rs12877718^4^ | rs7982639, rs10507382, rs17086497 |  |  |  |  |  |
|  |  |  | rs1324057^4^ | - |  |  |  |  |  |
|  |  |  | rs17086609 | - | 99 | 0.34 | 1 | 0.002 | 0.00137 |
|  |  |  | rs17086617 | rs2296284, rs7983774, rs9513095, rs1555641, rs9319428, rs9319429, rs7992068, rs9508025, rs9508026, rs9513105, rs9508029, rs10871266 | 96.5 | 0.26 | 1 | 0.721 | 0.50782 |
|  |  |  | rs2104330 | rs2296283, rs7332329, rs2057535, rs9551465, rs9319427, rs2182007, rs9551467, rs11149520, rs9943922 | 99.7 | 0.45 | 0.79 | 0.733 | 0.61062 |
|  |  |  | rs3751395 | - | 99.9 | 0.48 | 0.60 | 0.617 | 0.16356 |
|  |  |  | rs3751397 | - | 99.9 | 0.45 | 0.53 | 0.478 | 0.39418 |
|  |  |  | rs3936415 | - | 99.8 | 0.28 | 0.92 | 0.137 | 0.29840 |
|  |  |  | rs677471 | rs748253, rs679791, rs614820, rs612564, rs748252, rs600640 | 99.9 | 0.33 | 0.55 | 0.385 | 0.53352 |
|  |  |  | rs7330109 | rs9554330, rs9551471, rs7992940, rs718273, rs9551475 | 100 | 0.28 | 0.52 | 0.541 | 0.55427 |
|  |  |  | rs7987649 | - | 96.3 | 0.32 | 1 | 0.967 | 0.48820 |
|  |  |  | rs7995976^3^ | - |  |  |  |  |  |
|  |  |  | rs8002446 | rs9508033, rs9508034, rs722503, rs9319434, rs9513111 | 100 | 0.30 | 1 | 0.443 | 0.81292 |
|  |  |  | rs9319425 | - | 99.8 | 0.41 | 1 | 0.783 | 0.82498 |
|  |  |  | rs9508021 | rs7337610 | 99.9 | 0.34 | 0.14 | 0.491 | 0.34714 |
|  |  |  | rs9513070 | - | 99.9 | 0.41 | 0.46 | 0.443 | 0.65011 |
|  |  |  | rs9513089 | rs7339170, rs9513088, rs1853581, rs9508019 | 99.9 | 0.31 | 0.22 | 0.713 | 0.82857 |
|  |  |  | rs9513112 | rs2296285, rs9513113, rs9513116 | 100 | 0.30 | 0.17 | 0.705 | 0.67276 |
|  |  |  | rs9513114 | - | 99.8 | 0.28 | 0.66 | 0.735 | 0.45595 |
|  |  |  | rs9551462 | - | 99.8 | 0.30 | 0.75 | 0.324 | 0.09552 |
|  |  |  | rs9554320 | - | 99.9 | 0.43 | 0.72 | 0.077 | 0.00575 |
|  |  |  | rs9554322 | rs2387632 | 100 | 0.32 | 0.69 | 0.333 | 0.53414 |
|  |  |  | rs9554325 | - | 99.8 | 0.43 | 0.59 | 0.408 | 0.26479 |
|  |  |  | rs9579177^3^ | rs9579176, rs7993418, rs9554316, rs9554319, rs7982283, rs9513073, rs9513075 |  |  |  |  |  |
|  |  |  | rs9582036 | - | 99.9 | 0.30 | 0.03 | 0.140 | 0.01882 |
| *HES7* (1) | | 17p13.1 | rs1442849 | - | 99.6 | 0.29 | 0.04 | 0.900 | 0.73510 |
|  |  |  | rs3027232 | - | 99.9 | 0.21 | 0.49 | 0.207 | 0.77593 |
|  |  |  | rs3027279^4^ | rs8067165 |  |  |  |  |  |
| *HOXA1* (0.67) | | 7p15.3 | rs12700785 | - | 99.9 | 0.20 | 0.79 | 0.930 | 0.98901 |
|  |  |  | rs3807592 | - | 99.8 | 0.43 | 0.04 | 0.849 | 0.67219 |
| *HOXA2* (1) | | 7p15.2 | rs2428431 | - | 95.3 | 0.33 | 0.84 | 0.267 | 0.75884 |
| *HOXA3* (1) | | 7p15.2 | rs11764513^3^ | - |  |  |  |  |  |
|  |  |  | rs2428432 | - | 99.7 | 0.31 | 0.41 | 0.964 | 0.73586 |
|  |  |  | rs10085570 | rs4722657, rs7811753, rs4722659, rs2107063 | 99.8 | 0.15 | 1 | 0.767 | 0.88380 |
| *HOXA4* (0.75) | | 7p15.2 | rs4719884 | - | 99.9 | 0.22 | 0.52 | 0.839 | 0.58638 |
|  |  |  | rs6973182^3^ | rs11564055, rs2428433, rs2465276, rs7801565 |  |  |  |  |  |
|  |  |  | rs1859166 | - | 99.5 | 0.24 | 0.81 | 0.203 | 0.22718 |
| *HOXB1* (1) | | 17q21.3 | rs11079828^4^ | - |  |  |  |  |  |
|  |  |  | rs9905940 | rs35621842, rs12939811, rs8073963, rs8079617, rs8074125, rs7207109, rs6504340, rs2229302 | 100 | 0.22 | 1 | 0.634 | 0.44719 |
| *HOXB2* (1) | | 17q21.32 | rs1042815 | - | 99.7 | 0.40 | 0.24 | 0.502 | 0.10642 |
| *HOXB3* (1) | | 17q21.3 | rs999493 | - | 100 | 0.37 | 0.06 | 0.500 | 0.67523 |
|  |  |  | rs890432 | rs3826542 | 99.7 | 0.31 | 0.15 | 0.550 | 0.94628 |
|  |  |  | rs4793927^2^ | - |  |  |  |  |  |
|  |  |  | rs7503053^2^ | rs733920, rs6504389, rs11652148 |  |  |  |  |  |
|  |  |  | rs2740760^1^ | - |  |  |  |  |  |
|  |  |  | rs2740757 | rs2555110 | 99.8 | 0.16 | 0.12 | 0.361 | 0.49333 |
|  |  |  | rs2555111 | - | 99.9 | 0.48 | 0.01 | 0.228 | 0.83818 |
|  |  |  | rs2288277^2^ | rs7406324 |  |  |  |  |  |
| *HOXB4* (1) | | 17q21.32 | rs3809782^2^ | - |  |  |  |  |  |
| *HOXC4* (1) | | 12q13.3 | rs10747689 | rs894738, rs736825, rs894737, rs754133, rs4759318, rs10876528, rs10876529, rs10876530, rs2071449, rs12422600, rs765634 | 99.9 | 0.31 | 0.04 | 0.563 | 0.75435 |
|  |  |  | rs10876532 | rs2171216 | 99.8 | 0.41 | 0.09 | 0.470 | 0.33363 |
|  |  |  | rs11170792 | rs11608918 | 99.9 | 0.2 | 0.78 | 0.531 | 0.29813 |
|  |  |  | rs11615193^4^ | - |  |  |  |  |  |
|  |  |  | rs2630772 | - | 99.6 | 0.27 | 0.73 | 0.817 | 0.77290 |
|  |  |  | rs4759317 | rs11170786 | 99.6 | 0.41 | 0.05 | 0.469 | 0.86481 |
|  |  |  | rs7136731 | rs10747691, rs10747692, rs1386016 | 100 | 0.39 | 0.14 | 0.816 | 0.97287 |
|  |  |  | rs7297416 | rs4759062, rs7136889, rs7315688, rs6580970, rs10876531 | 99.9 | 0.26 | 0.03 | 0.616 | 0.65858 |
|  |  |  | rs746423 | - | 99.9 | 0.16 | 0.87 | 0.663 | 0.99276 |
| *HOXD1* (1) | | 2q31.1 | rs6433572 | - | 99.9 | 0.43 | 0.18 | 0.926 | 0.80795 |
| *HOXD3* (1) | | 2q31.1 | rs717852 | rs2857540, rs711830, rs1318778, rs2072590 | 100 | 0.30 | 1 | 0.453 | 0.23507 |
|  |  |  | rs10834 | - | 99.5 | 0.32 | 0.47 | 0.203 | 0.10622 |
|  |  |  | rs7600360^3^ | - |  |  |  |  |  |
|  |  |  | rs13028842 | - | 99.8 | 0.28 | 0.83 | 0.654 | 0.41450 |
|  |  |  | rs1374325 | rs9141, rs34727427, rs7606110, rs2301301, rs13017511, rs6759499, rs13390932 | 100 | 0.34 | 0.63 | 0.785 | 0.92940 |
| *HOXD4* (0.67) | | 2q31.1 | rs4972504^1^ | - |  |  |  |  |  |
|  |  |  | rs4972505 | rs4972806, rs1867863, rs2071581, rs1542180, rs4246626, rs1446575 | 98.2 | 0.36 | 0.77 | 0.605 | 0.33873 |
| *ID1* (1) | | 20q11 | rs6060262^4^ | rs6060260, rs6060261, rs15817, rs6058189, rs6058197 |  |  |  |  |  |
| *ID2* (1) | | 2p25 | rs35434759 | - | 99.8 | 0.12 | 0.66 | 0.271 | 0.33984 |
|  |  |  | rs4669330 | - | 99 | 0.25 | 0.34 | 0.240 | 0.58572 |
| *KDR* (1) | | 4q11-q12 | rs10020464 | - | 99.9 | 0.30 | 0.13 | 0.024 | 0.42635 |
|  |  |  | rs11732292 | rs7655964, rs6848933, rs2168945, rs13109660, rs2305945, rs12507807, rs3828550 | 99.6 | 0.32 | 0.37 | 0.648 | 0.28622 |
|  |  |  | rs12502008 | - | 100 | 0.34 | 0.63 | 0.322 | 0.72294 |
|  |  |  | rs12642307 | - | 100 | 0.27 | 1 | 0.574 | 0.57636 |
|  |  |  | rs1531289 | - | 99.8 | 0.32 | 0.60 | 0.105 | 0.14734 |
|  |  |  | rs1531290 | - | 99.9 | 0.50 | 0.79 | 0.299 | 0.35548 |
|  |  |  | rs1551641 | rs1551642 | 100 | 0.31 | 0.68 | 0.627 | 0.75421 |
|  |  |  | rs17709898 | rs7671745 | 100 | 0.35 | 0.77 | 0.367 | 0.80718 |
|  |  |  | rs2034965 | - | 100 | 0.26 | 0.08 | 0.426 | 0.06085 |
|  |  |  | rs2071559 | rs7667298 | 99.6 | 0.49 | 0.38 | 0.140 | 0.65601 |
|  |  |  | rs2239702^4^ | - |  |  |  |  |  |
|  |  |  | rs6828477 | rs7673274, rs7654599 | 99.6 | 0.44 | 0.15 | 0.281 | 0.34831 |
|  |  |  | rs6838752^3^ | rs17085265, rs2219471, rs1870377, rs1870379, rs1870378, rs13136007, rs17085262 |  |  |  |  |  |
|  |  |  | rs7692791 | rs11133360 | 99.8 | 0.45 | 0.79 | 0.325 | 0.65374 |
| *LFNG* (1) | | 7p22.2 | rs11770785 | - | 94.9 | 0.34 | 0.01 | 0.554 | 0.30340 |
|  |  |  | rs13234810 | - | 96.4 | 0.38 | 0.13 | 0.807 | 0.88176 |
|  |  |  | rs1982157^4^ | - |  |  |  |  |  |
|  |  |  | rs2895 | - | 100 | 0.10 | 0.21 | 0.023 | 0.07548 |
|  |  |  | rs4632959 | - | 99.6 | 0.49 | 0.01 | 0.942 | 0.40097 |
|  |  |  | rs755179 | - | 100 | 0.48 | 0.03 | 0.277 | 0.64238 |
| *MESP2* (0.50) | | 15q26.1 | rs12908026 | rs12900413, rs11632886 | 99.5 | 0.26 | 0.82 | 0.654 | 0.58699 |
|  |  |  | rs6496601 | rs6496602, rs12908343 | 99.9 | 0.30 | 0.60 | 0.905 | 0.86206 |
| *Msgn1* (1) | | 2p24.2 | rs11689375 | - | 99.5 | 0.44 | 0.29 | 0.012 | 0.00810 |
|  |  |  | rs6711333 | rs4132753, rs4432452, rs4075922 | 99.9 | 0.50 | 0.06 | 0.068 | 0.04409 |
|  |  |  | rs6754626 | - | 100 | 0.46 | 0.54 | 0.292 | 0.12409 |
| *NF1* (1) | | 17q11.2 | rs11080149^3^ | - |  |  |  |  |  |
|  |  |  | rs12943508 | rs2952998, rs1123232, rs4239229, rs4263003, rs4559963, rs7503395, rs4541129, rs12948444, rs11870097, rs7220268, rs7212264, rs11868459, rs4795579, rs7214792, rs12949230, rs12942479, rs12942397, rs12603885, rs2905794, rs2952982, rs2952980, rs2952978, rs2905790, rs2905788, rs2269855, rs2952976, rs1124918, rs2953013, rs2953012, rs2953009, rs1801052, rs2953000, rs2952999, rs2905878, rs2952989, rs2905869, rs2905804, rs2905803, rs2905870, rs2905801, rs2905872, rs2952991, rs1013946, rs2905800, rs2285812, rs2341348, rs1034705, rs2952993, rs2952995, rs2905875, rs2905877, rs2905880, rs984125, rs2012988, rs2905795, rs2905881, rs2285892, rs8067021, rs2072131, rs2066736, rs2018634, rs2018624, rs2012581, rs9894862, rs9913470, rs9898664, rs9896041, rs7216033, rs7220483, rs9303642, rs11080146, rs4795587, rs9906488, rs7406870, rs757384, rs10512433, rs8080679, rs12945157, rs12940303, rs11080148, rs4795591, rs4795593, rs9892839, rs9902427, rs9902893, rs2040791, rs11080150, rs3087591, rs12940802, rs7226006, rs6505234, rs2057769, rs2107360, rs2854322, rs2051507, rs7406983, rs1800845, rs7210604 | 99.6 | 0.25 | 0.82 | 0.515 | 0.13149 |
|  |  |  | rs2905873 | rs7215555 | 99.9 | 0.28 | 0.74 | 0.628 | 0.80062 |
|  |  |  | rs2905874 | rs1013948, rs2952975, rs2905808, rs3815156 | 100 | 0.14 | 0.14 | 0.898 |  |
|  |  |  | rs2953014 | - | 100 | 0.22 | 0.90 | 0.907 |  |
|  |  |  | rs2953016 | - | 98.8 | 0.20 |  | 0.348 | 0.04996 |
|  |  |  | rs3815154 | rs7505, rs1129506, rs3785956, rs2525563, rs2854306, rs35109242, rs2854307, rs2525564, rs9894648, rs2285894, rs7503922, rs2854308, rs2525565, rs2189525, rs2854311, rs2525568, rs2525569, rs964288, rs7350943, rs7502834, rs7406783, rs10438801, rs2854319, rs2854320, rs2107359, rs1048317, rs8067440, rs2040792, rs2214538, rs10512434, rs7218930, rs2342319, rs12943365, rs2525570, rs2525574 | 99.9 | 0.38 | 0.51 | 0.493 | 0.25235 |
| *NKD1* (0.86) | 16q12.1 | rs12596811 | - | 99.8 | 0.22 | 0.13 | 0.285 | 0.54268 |  |
|  |  | rs4785220 | - | 100 | 0.35 | 0.70 | 0.373 | 0.36972 |  |
|  |  | rs7204135 | rs4785438, rs7203943 | 100 | 0.47 | 0.04 | 0.113 | 0.44195 |  |
|  |  | rs8047222 | rs8046845, rs7206907, rs1558663, rs11640716, rs7198686, rs12913, rs745230 | 100 | 0.38 | 0.41 | 0.164 | 0.15879 |  |
|  |  | rs933566 | - | 100 | 0.47 | 0.38 | 0.064 | 0.35890 |  |
|  |  | rs9673419 | - | 99.9 | 0.30 | 0.19 | 0.052 | 0.06422 |  |
| *NKD2* (1) | 5p15.3 | rs10040568 | rs881334, rs4975577, rs6876150 | 99.5 | 0.42 | 0.65 | 0.168 | 0.13449 |  |
|  |  | rs12517757 | rs4975576 | 99.9 | 0.48 | 0.93 | 0.012 | 0.02614 |  |
|  |  | rs16870629 | - | 100 | 0.46 | 0.72 | 0.841 | 0.96584 |  |
|  |  | rs4975528^4^ | - |  |  |  |  |  |  |
| *NOG* (0.75) | 17q22 | rs1442828 | - | 99.7 | 0.30 | 0.37 | 0.022 | 0.13493 |  |
|  |  | rs16957413 | - | 100 | 0.12 | 0.24 | 0.053 | 0.74653 |  |
|  |  | rs9915822 | - | 100 | 0.49 | 0.73 | 0.483 | 0.91409 |  |
| *NOTCH1* (0.86) | 9q34.3 | rs10870079^4^ | - |  |  |  |  |  |  |
|  |  | rs11145760 | - | 99.3 | 0.42 | 0.86 | 0.647 | 0.92578 |  |
|  |  | rs11145770 | - | 100 | 0.34 | 0.10 | 0.736 | 0.46303 |  |
|  |  | rs13290979 | - | 99.6 | 0.34 | 0.33 | 0.545 | 0.19021 |  |
|  |  | rs2229971 | - | 99.6 | 0.27 | 0.59 | 0.263 | 0.24443 |  |
|  |  | rs2229974^4^ | - |  |  |  |  |  |  |
|  |  | rs3013304 | rs3013302, rs3013300 | 99.1 | 0.29 | 0.83 | 0.546 | 0.66832 |  |
|  |  | rs3013309 | - | 99.8 | 0.29 | 0.46 | 0.897 | 0.03351 |  |
|  |  | rs3124596^4^ | rs3124594 |  |  |  |  |  |  |
|  |  | rs3124603 | rs3125009, rs3124607 | 99.3 | 0.32 | 0.07 | 0.382 | 0.20360 |  |
|  |  | rs3124999 | rs10521 | 98.9 | 0.34 | 0.62 | 0.901 | 0.99079 |  |
|  |  | rs3125002^4^ | - |  |  |  |  |  |  |
|  |  | rs3812604 | - | 99.8 | 0.41 | 0.86 | 0.691 | 0.83829 |  |
|  |  | rs3812605 | rs3812603 | 97.7 | 0.46 | 0.86 | 0.975 | 0.79962 |  |
|  |  | rs4489420^4^ | - |  |  |  |  |  |  |
|  |  | rs6563 | - | 99.7 | 0.45 | 0.18 | 0.334 | 0.37152 |  |
|  |  | rs710411 | - | 99.8 | 0.37 | 0.85 | 0.749 | 0.59390 |  |
|  |  | rs9314867 | - | 100 | 0.45 | 0.72 | 0.272 | 0.04188 |  |
|  |  | rs9411255 | - | 100 | 0.28 | 0.30 | 0.080 | 0.04052 |  |
| *PRRX1* (1) | 1q24 | rs10489232^3^ | - |  |  |  |  |  |  |
|  |  | rs10919449 | - | 99.9 | 0.41 | 0.41 | 0.444 | 0.61853 |  |
|  |  | rs12566345 | rs12744534, rs2421490, rs2213751 | 100 | 0.11 | 0.83 | 0.589 | 0.38478 |  |
|  |  | rs17551172 | rs17551016, rs736022, rs4656222, rs2421491, rs12409048, rs1016819, rs17550940, rs4656796, rs17551291, rs17551298, rs3820416 | 100 | 0.29 | 0.40 | 0.877 | 0.62052 |  |
|  |  | rs4656220 | rs4656794 | 99.9 | 0.35 | 0.12 | 0.976 | 0.82686 |  |
|  |  | rs4656797 | rs489090, rs2187895, rs6701640 | 100 | 0.20 | 1 | 0.531 | 0.49467 |  |
|  |  | rs4656799 | - | 100 | 0.38 | 0.05 | 0.535 | 0.43676 |  |
|  |  | rs502612 | - | 100 | 0.46 | 0.03 | 0.291 | 0.28488 |  |
|  |  | rs531650 | rs1476040 | 99.1 | 0.29 | 0.83 | 0.577 | 0.56812 |  |
|  |  | rs541618^4^ | rs513287, rs10800531 |  |  |  |  |  |  |
|  |  | rs591715 | - | 99.9 | 0.22 | 0.45 | 0.873 | 0.59205 |  |
|  |  | rs593479^1^ | - |  |  |  |  |  |  |
|  |  | rs600093 | rs520525 | 100 | 0.29 | 0.67 | 0.583 | 0.54736 |  |
|  |  | rs659580 | - | 99 | 0.23 | 0.70 | 0.230 | 0.17715 |  |
|  |  | rs662871 | rs7520192, rs520131 | 100 | 0.39 | 0.58 | 0.642 | 0.47434 |  |
|  |  | rs7540713 | - | 99.6 | 0.30 | 0.40 | 0.841 | 0.33940 |  |
| *RARA* (1) | 17q21 | rs2715553 | - | 100 | 0.44 | 0.86 | 0.790 | 0.88317 |  |
|  |  | rs2715554 | - | 99.7 | 0.13 | 0.56 | 0.971 | 0.95429 |  |
|  |  | rs482284 | - | 98.4 | 0.31 | 0.32 | 0.023 | 0.11733 |  |
|  |  | rs9303286 | rs7217852, rs9904270, rs12946680, rs9303285 | 99.1 | 0.11 | 0.66 | 0.738 | 0.68453 |  |
| *RARG* (0.75) | 12q13 | rs1554753 | - | 100 | 0.20 | 1 | 0.007 | 0.24999 |  |
|  |  | rs3741434 | - | 98.5 | 0.13 | 0.70 | 0.799 | 0.64854 |  |
|  |  | rs6580936 | - | 100 | 0.15 | 1 | 0.008 | 0.01752 |  |
| *RBP1* (1) | 3q23 | rs12485273 | rs2071387 | 99.9 | 0.14 | 0.38 | 0.265 | 0.24278 |  |
|  |  | rs184157 | rs184156 | 100 | 0.26 | 0.91 | 0.875 | 0.57743 |  |
|  |  | rs186105 | - | 99.8 | 0.41 | 0.21 | 0.228 | 0.49727 |  |
|  |  | rs190910 | rs10935331, rs893704, rs176990 | 98.7 | 0.42 | 0.01 | 0.520 | 0.60140 |  |
|  |  | rs2071388 | rs176987, rs188420, rs188421, rs295484, rs167187 | 98.8 | 0.40 | 0.12 | 0.918 | 0.98941 |  |
|  |  | rs211585 | rs295486, rs188422, rs295488 | 99.7 | 0.49 | 0.11 | 0.752 | 0.71110 |  |
|  |  | rs295490 | rs397940 | 100 | 0.15 | 1 | 0.478 | 0.74983 |  |
|  |  | rs295492^4^ | rs190909, rs295493 |  |  |  |  |  |  |
|  |  | rs747346 | rs9834607, rs9835241, rs13086868 | 100 | 0.22 | 0.16 | 0.901 | 0.55793 |  |
|  |  | rs893703^3^ | rs4683502, rs3772868, rs3821543, rs4407373, rs1511570, rs12493507 |  |  |  |  |  |  |
|  |  | rs9821204 | - | 99.2 | 0.23 | 0.28 | 0.589 | 0.56187 |  |
| *RBP4* (1) | 10q23-q24 | rs10882273 | rs10882272 | 99.3 | 0.40 | 0.58 | 0.745 | 0.87945 |  |
|  |  | rs11187537 | rs11187536 | 99.5 | 0.28 | 0.28 | 0.711 | 0.57526 |  |
|  |  | rs17108978 | - | 99.2 | 0.32 | 0.84 | 0.244 | 0.83092 |  |
|  |  | rs34571439 | rs12766992, rs17108973, rs13376835, rs17108991, rs17484721 | 98.5 | 0.22 | 1 | 0.478 | 0.81908 |  |
|  |  | rs7079946 | - | 98.7 | 0.27 | 0.57 | 0.787 | 0.66965 |  |
|  |  | rs7094671 | - | 99.8 | 0.29 | 0.91 | 0.301 | 0.91472 |  |
| *RBPJ1* (1) | 4p15.2 | rs946346^3^ | - |  |  |  |  |  |  |
|  |  | rs13109703 | rs13114911, rs13133397, rs2725303, rs2725304, rs11722744, rs2725309, rs13128014, rs2725305, rs13119840, rs4289434, rs4692532, rs11727747, rs4536914, rs7655165, rs2725325, rs13115260, rs3109843, rs3109842, rs11726853, rs3109839, rs7349722, rs13147671, rs3113016, rs3109849, rs12639629, rs2788864, rs2270225, rs10939110, rs10939111 | 99.3 | 0.45 | 0.86 | 0.121 | 0.43649 |  |
|  |  | rs17639483 | rs1877207, rs13109842, rs13128399, rs13135696, rs3762930, rs2063446 | 98.8 | 0.46 | 0.72 | 0.239 | 0.40327 |  |
|  |  | rs3109836 | - | 100 | 0.17 | 0.30 | 0.015 | 0.15916 |  |
|  |  | rs3796802^4^ | - |  |  |  |  |  |  |
|  |  | rs6816542 | - | 99.8 | 0.12 | 0.28 | 0.431 | 0.38265 |  |
|  |  | rs6821126 | rs4330343 | 100 | 0.19 | 1 | 0.935 | 0.89911 |  |
|  |  | rs6853254 | rs2153155, rs3109828, rs3109841 | 99.9 | 0.36 | 0.28 | 0.065 | 0.23727 |  |
| *RDH10* (1) | 8q21.11 | rs10110749^4^ | - |  |  |  |  |  |  |
|  |  | rs11776584 | rs7827088, rs11774608, rs4374985, rs17287095, rs17214921, rs11779182, rs17287304 | 99.9 | 0.24 | 0.72 | 0.790 | 0.51517 |  |
|  |  | rs11779605 | rs10957652, rs6989495, rs3793373 | 99.8 | 0.32 | 0.13 | 0.881 | 0.52753 |  |
|  |  | rs16938610 | rs4737370 | 100 | 0.22 | 0.15 | 0.022 | 0.00903 |  |
|  |  | rs2070764 | - | 99.9 | 0.31 | 0.55 | 0.034 | 0.02789 |  |
|  |  | rs4394387 | rs4237000 | 99.3 | 0.25 | 0.54 | 0.169 | 0.11285 |  |
|  |  | rs6472765 | rs4237001, rs7840840, rs10957653, rs4738319, rs4738320 | 99.5 | 0.26 | 0.48 | 0.230 | 0.09481 |  |
|  |  | rs7837090 | rs7819550, rs9886504 | 99.8 | 0.20 | 0.25 | 0.384 | 0.95478 |  |
|  |  | rs7843902 | - | 99.2 | 0.46 | 0.79 | 0.126 | 0.21713 |  |
| *RXRA* (1) | 9q34.3 | rs11103473 | - | 99.9 | 0.39 | 0.85 | 0.899 | 0.76198 |  |
|  |  | rs1805352 | rs3132296, rs3118529, rs1887309 | 99.3 | 0.30 | 0.29 | 0.225 | 0.71384 |  |
|  |  | rs3118571 | rs877954, rs1805343, rs4842194 | 99.7 | 0.36 | 0.33 | 0.097 | 0.34123 |  |
|  |  | rs4240705 | - | 100 | 0.37 | 0.51 | 0.304 | 0.28401 |  |
|  |  | rs4842196 | - | 99.9 | 0.26 | 0.91 | 0.297 | 0.55818 |  |
|  |  | rs875444 | - | 99.9 | 0.44 | 0.53 | 0.965 | 0.67358 |  |
| *SHH* (1) | 7q36 | rs1233556 | - | 99.9 | 0.15 | 0.40 | 0.578 | 0.44141 |  |
|  |  | rs1233560^4^ | - |  |  |  |  |  |  |
|  |  | rs1233571 | - | 99.6 | 0.15 | 0.28 | 0.336 | 0.39889 |  |
|  |  | rs2239966 | - | 93.8 | 0.39 | 0.71 | 0.289 | 0.23116 |  |
|  |  | rs288746 | - | 100 | 0.13 | 0.45 | 0.726 | 0.36522 |  |
|  |  | rs872723 | - | 100 | 0.25 | 0.21 | 0.253 | 0.22327 |  |
| *SNAI1* (1) | 20q13.2 | rs1543442 | - | 98.3 | 0.41 | 0.85 | 0.790 | 0.80534 |  |
|  |  | rs16995010 | rs6095728, rs6020166, rs4647958, rs4647957, rs16995009, rs4647959, rs6091078, rs6020177, rs6020179 | 98.5 | 0.14 | 0.71 | 0.339 | 0.91906 |  |
|  |  | rs6020170 | rs6091080, rs6020178, rs6012791 | 99.9 | 0.21 | 1 | 0.764 | 0.38425 |  |
|  |  | rs6125849^4^ | - |  |  |  |  |  |  |
| *SNAI2* (1) | 8q11 | rs11781122 | rs6982389 | 99.9 | 0.38 | 0.23 | 0.778 | 0.70602 |  |
|  |  | rs1992375 | - | 99.8 | 0.50 | 0.86 | 0.418 | 0.37442 |  |
|  |  | rs2735455^3^ | - |  |  |  |  |  |  |
| *STAT3* (1) | 17q21.31 | rs1053004 | - | 99.8 | 0.39 | 1 | 0.803 | 0.57457 |  |
|  |  | rs12601982 | rs2293155, rs8078731, rs2293154, rs3198502, rs1053023, rs1053005, rs3744483, rs8074524, rs3809758, rs9909659 | 100 | 0.16 | 1 | 0.079 | 0.04114 |  |
|  |  | rs12949918^1^ | rs17885629, rs744166, rs12942547 |  |  |  |  |  |  |
|  |  | rs17593222^3^ | - |  |  |  |  |  |  |
|  |  | rs2293152^4^ | - |  |  |  |  |  |  |
|  |  | rs6503695 | - | 99.5 | 0.3 | 0.59 | 0.129 | 0.06506 |  |
|  |  | rs7217655 | rs3816769, rs957970, rs1026916, rs4796791, rs7219739, rs9891119, rs7211777 | 100 | 0.35 | 0.77 | 0.181 | 0.06357 |  |
|  |  | rs8069645 | rs6503696, rs6503697, rs4103200, rs9912773, rs3785898, rs4796793 | 100 | 0.26 | 0.64 | 0.169 | 0.07175 |  |
| *STRA6* (1) | 15q24.1 | rs10910 | rs1052622 | 99.8 | 0.26 | 0.43 | 0.412 | 0.58514 |  |
|  |  | rs11633768 | - | 100 | 0.28 | 0.19 | 0.451 | 0.70832 |  |
|  |  | rs11855667 | - | 99.7 | 0.18 | 0.76 | 0.669 | 0.35794 |  |
|  |  | rs12915891 | rs3888004, rs13259, rs3848161, rs2899824, rs11630317, rs11631944, rs12912578, rs409856, rs12899173, rs971757, rs971756, rs971755, rs11857410, rs733163, rs12912258, rs11639369, rs11630924 | 99.9 | 0.15 | 1 | 0.602 | 0.35978 |  |
|  |  | rs351219 | - | 100 | 0.37 | 0.13 | 0.504 | 0.64228 |  |
|  |  | rs351222^1^ | - |  |  |  |  |  |  |
|  |  | rs351224 | rs351223 | 100 | 0.48 | 0.48 | 0.872 | 0.90397 |  |
|  |  | rs351238 | rs351237 | 99.1 | 0.34 | 1 | 0.875 | 0.60930 |  |
|  |  | rs974456 | - | 100 | 0.20 | 0.79 | 0.809 | 0.94518 |  |
| *T* (1) | 6q27 | rs1056053^1^ | rs1134481 |  |  |  |  |  |  |
|  |  | rs12208710 | rs13191186, rs3127328, rs12207298, rs2277094, rs3816304, rs12154075, rs12200529, rs2028792, rs2028791 | 100 | 0.11 | 0.68 | 0.160 | 0.40843 |  |
|  |  | rs1867592 | - | 99.9 | 0.10 | 0.82 | 0.201 | 0.35923 |  |
|  |  | rs1867594 | rs10946129, rs12207747, rs12207966, rs16898752, rs12215543, rs10946130 | 99.9 | 0.28 | 0.75 | 0.646 | 0.43997 |  |
|  |  | rs3099266 | rs2197450 | 99.8 | 0.41 | 0.58 | 0.260 | 0.86018 |  |
|  |  | rs3099282 | rs6928216, rs11962977, rs1122751 | 99.8 | 0.22 | 0.17 | 0.294 | 0.68028 |  |
|  |  | rs3127334 | rs1056051, rs3127329, rs3127331, rs3099268, rs3127332, rs3099270, rs3127345 | 99.9 | 0.28 | 0.91 | 0.075 | 0.73747 |  |
|  |  | rs3127344 | rs2305089 | 100 | 0.48 | 0.79 | 0.209 | 0.73159 |  |
|  |  | rs6917533 | rs9356431, rs6914547, rs9356430, rs3127337 | 100 | 0.43 | 0.79 | 0.228 | 0.36232 |  |
|  |  | rs7773665 | - | 100 | 0.38 | 0.78 | 0.953 | 0.63724 |  |
|  |  | rs9356434 | - | 99.9 | 0.30 | 0.92 | 0.571 | 0.54778 |  |
|  |  | rs9459589 | rs9295321, rs9295322 | 100 | 0.33 | 0.92 | 0.076 | 0.42514 |  |
|  |  | rs9459593^1^ | rs6927011, rs6931915, rs9295323 |  |  |  |  |  |  |
| *TBX6* (1) | 16p11.2 | rs11644459 | rs8060511 | 98.2 | 0.44 | 0.65 | 0.685 | 0.49640 |  |
|  |  | rs11861842^3^ | rs11863129 |  |  |  |  |  |  |
|  |  | rs2278557 | rs28592059 | 99.9 | 0.34 | 0.29 | 0.503 | 0.61958 |  |
|  |  | rs2289292^1^ | - |  |  |  |  |  |  |
|  |  | rs3809624 | - | 99.9 | 0.33 | 0.76 | 0.735 | 0.73654 |  |
| *VEGFA* (1) | 6p12 | rs10434 | - | 97.5 | 0.46 | 0.21 | 0.083 | 0.22286 |  |
|  |  | rs2146323 | rs3025010 | 100 | 0.33 | 0.28 | 0.912 | 0.99429 |  |
|  |  | rs25648 | - | 99.1 | 0.16 | 0.63 | 0.649 | 0.91608 |  |
|  |  | rs3025000 | rs833060, rs833068, rs833069, rs3024997 | 99.1 | 0.27 | 0.19 | 0.271 | 0.44600 |  |
|  |  | rs3025030 | rs3025033 | 100 | 0.12 | 1 | 0.453 | 0.84163 |  |
|  |  | rs3025040 | - | 100 | 0.13 | 1 | 0.773 | 0.68231 |  |
|  |  | rs6899540 | - | 99.7 | 0.16 | 0.53 | 0.327 | 0.23270 |  |
|  |  | rs6900017^3^ | - |  |  |  |  |  |  |
|  |  | rs833070 | rs833061, rs699947 | 100 | 0.48 | 0.66 | 0.805 | 0.33946 |  |
|  |  | rs998584 | - | 99.8 | 0.47 | 0.38 | 0.111 | 0.04011 |  |
| *WNT3A* (1) | 1q42 | rs10916258 | - | 99.8 | 0.21 | 1 | 0.680 | 0.96261 |  |
|  |  | rs13374948^3^ | rs6672559, rs6672422, rs7539664, rs1034792, rs10916262, rs11589513, rs11584499, rs882451, rs4653533 |  |  |  |  |  |  |
|  |  | rs1636195 | rs697763, rs708118, rs708121, rs3094911, rs708109, rs1745413, rs708123, rs708124, rs708108, rs697761, rs697762, rs708113 | 99.8 | 0.34 | 0.92 | 0.978 | 0.77371 |  |
|  |  | rs1745423^4^ | - |  |  |  |  |  |  |
|  |  | rs3094912 | rs708112, rs964941, rs708114, rs3094914, rs3094913, rs708111, rs947631 | 100 | 0.44 | 0.59 | 0.430 | 0.78434 |  |
|  |  | rs3121309^4^ | - |  |  |  |  |  |  |
|  |  | rs708122 | - | 100 | 0.35 | 0.77 | 0.227 | 0.66144 |  |
|  |  | rs766972 | rs3121310, rs752107 | 98.9 | 0.31 | 1 | 0.722 | 0.83730 |  |
|  |  |  |  |  |  |  |  |  |  |
|  |  |  |  |  |  |  |  |  |  |
|  |  |  |  |  |  |  |  |  |  |
| Abbreviations: MAF, Minor Allele Frequency | | |  |  |  |  |  |  |  |
|  |  |  |  |  |  |  |  |  |  |
| SNP^n^: Excluded for the following reasons: ^1^TagSNP in Linkage Disequilibrium with another one across the gene in our sample of 350 controls; ^2^Not in Hardy-Weinberg equilibrium in the control sample (p<0.01); ^3^ Monomorphic or MAF<0.10; ^4^Genotyping Failure; ^5^Not passing through the SNPlex design pipeline | | | | | | | |  |  |
|  |  |  |  |  |  |  |  |  |  |
|  |  |  |  |  |  |  |  |  |  |
| SNP^&^: Non-synonymous SNPs additionally included in the tagSNPs list. | | | |  |  |  |  |  |  |
|  |  |  |  |  |  |  |  |  |  |
| ^a^ Gene Coverage= tag SNPs analyzed/total of tag SNPs per gene | | | |  |  |  |  |  |  |
| ^b^ According to HAPMAP Data Rel 27 PhaseII+III, Feb09, on NCBI B36 assembly, dbSNP b126 | | | |  |  |  |  |  |  |
| ^c^ MAF of cases and controls | |  |  |  |  |  |  |  |  |
| ^d^ HWE test performed in the control sample ) | | |  |  |  |  |  |  |  |
| ^e^ p-value calculated using the Cochran-Armitage Trend Test (ATT | | | |  |  |  |  |  |  |
